# Comparison of deep learning-based three-dimensional human pose estimation methods with motion capture for gesture research

**DOI:** 10.1371/journal.pone.0347288

**Published:** 2026-04-24

**Authors:** Naoto Ienaga, Kazuki Sekine

**Affiliations:** 1 Institute of Systems and Information Engineering, University of Tsukuba, Tsukuba, Ibaraki, Japan; 2 Faculty of Human Sciences, Waseda University, Tokorozawa, Saitama, Japan; Indian Institute of Technology Patna, INDIA

## Abstract

Humans often make gestures while speaking. These gestures have been extensively researched in psychology and cognitive science, revealing their functions and roles in communication. Despite being produced in three-dimensional (3D) space, gestures and gesture space have primarily been measured and analyzed using two-dimensional planes. Optical motion capture (MoCap) is commonly used for 3D gesture measurement. However, MoCap is expensive, requires a large space, and the attachment of retroreflective markers can interfere with the natural generation of gestures by the speaker. Recent advances in deep learning-based human pose estimation (HPE) offer promising alternatives by enabling 3D keypoint estimation from standard video cameras. This study investigates the accuracy of four HPEs—two monocular and two stereo with triangulation methods—in estimating upper-body keypoints commonly used in natural scenarios for gesture research. Ten participants were recorded performing gesture-rich speech by both a MoCap system and three video cameras. We compared 13 keypoints, including the wrists, elbows, shoulders, fingers, and face, with MoCap data, using Euclidean distance as an error metric. Statistical analyses revealed that stereo methods significantly outperformed monocular methods across all keypoints. The most accurate method achieved an average error of 49.4 mm with respect to MoCap, suggesting sufficient accuracy for practical gesture analysis. Additionally, we visualized 3D gesture spaces and found a 75.4% overlap between HPE and MoCap at a voxel size of 50 mm, indicating high spatial agreement. Our findings demonstrate that stereo HPE methods are viable, cost-effective alternatives to MoCap in gesture-related applications. Our ultimate objective is to create a toolbox that is accurate and easy to use for measuring human gestures in 3D space by exploiting the recent advances in HPE as an alternative to MoCap. Such a toolbox will be suitable for non-experts in machine learning, and these results lay the foundation for it.

## Introduction

Research on gestures has clarified their functions and roles in communication and has contributed significantly to fields such as psychology and cognitive science. Although body movements are performed in three-dimensional (3D) space, most studies have traditionally relied on two-dimensional (2D) analysis. However, this approach has limitations. The depth axis (the front-back direction) of gestures has been shown to carry important information in contexts such as dialogue and learning [1]. Previous research has shown that 3D estimation methods, including those based on depth sensors such as the Microsoft Kinect, capture spatial features of gestures more accurately than 2D analysis alone [2–5]. Thus, although 2D analyses remain useful in many contexts, empirical evidence indicates that 3D estimation offers a more comprehensive account of gesture space. Additionally, “gesture space”, which is the spatial area in which gestures are performed, is an important medium for information transmission. The concept of gesture space was first systematically described by [6] as part of his foundational framework on gesture-speech relations. Although subsequent research has moved beyond this framework, it remains one of the most influential starting points for analyzing gesture space (see also [1,4,7]). However, this simplification overlooks critical 3D dynamics.

Optical motion capture (MoCap) is a conventional method for measuring body movements in 3D space. MoCap has a high accuracy and frame rate but it has three significant drawbacks: (1) It is expensive and can be used only in a few research institutions that have abundant research funding. In addition to the cost of its hardware, the high cost of its software, which must be regularly updated, is a concern. (2) A large space is required because of the size of the equipment and the time-consuming setup process. In certain situations, MoCap may require a dedicated space. (3) The retroreflective markers necessary for MoCap measurements can interfere with the generation of natural gestures by the speaker. In fact, participants in this study were observed to be paying attention to the markers on their fingers, despite being instructed to gesture as naturally as possible. This poses a significant problem in studies of natural gestures.

Recent advances in artificial intelligence, particularly in deep learning, have led to remarkable progress in human pose estimation (HPE), which is a technique for identifying human body keypoints from images or video [8]. Keypoints are points of interest on the human body, and they mainly consist of joints such as the wrists, elbows, and shoulders. There are also keypoints on the face; for example, the contours of the face, lips, and eyes. Early HPE methods estimated only the 2D positions of the keypoints of a single person. However, recent advances have made it possible to estimate the keypoints of multiple people and the 3D positions of the keypoints from a single viewpoint quickly and accurately. These advances have made HPE quite practical.

Our study focuses on the accuracy of HPE for analyzing existing movements. A related and rapidly advancing field is human motion prediction and generation, which aims to synthesize realistic future motions. These approaches often use spatio-temporal models, such as graph convolutional networks or diffusion transformers, to generate realistic human animations [9–11]. The success of these generative tasks fundamentally depends on the quality and accuracy of the underlying pose data. Therefore, our study’s foundational analysis of HPE accuracy remains crucial for advancing these related applications.

Regarding the accuracy of these estimation techniques, gait parameters, such as the step time and step length of participants, have been estimated using OpenPose [12] from videos recorded simultaneously with MoCap [13]. The estimated parameters were compared with those of MoCap. Although OpenPose was able to produce values similar to those of MoCap for movements that were mostly confined to a 2D plane, the error in the depth direction was substantial. Another study compared the accuracies of 3D keypoint estimation of OpenPose, AlphaPose [14], and DeepLabCut [15] against MoCap using a video camera system capable of recording at 200 Hz with nine cameras [16]. That study also emphasized the high accuracy of deep learning-based methods. However, the video camera system used in the study was quite large and expensive, albeit less large and expensive than that required by MoCap. In addition, the movements analyzed in that study were simple; for example, walking, running, and jumping. The accuracy of OpenPose has also been verified for more complex movements. In a study that compared OpenPose with MoCap using five video cameras, the error was reported to be 30 mm or less for several movements, including throwing, which is a fast 3D motion [17]. Another study reported an average error of less than 70 mm when keypoints were estimated during pop dance from four videos [18].

As reviewed above, the practical application of the 3D estimation of keypoints using multiple cameras and HPE is feasible. However, previous studies have the following problems:

- Application to complex movements, such as throwing and dancing, has been attempted, but verification from the perspective of gesture research has been insufficient. HPE has also been used in the context of gesture research, but several of these studies were limited to 2D analysis [7,19]). However, 2D based methods often fall short in capturing the full spatial nuance of gestures. A shift toward high-quality 3D estimation methods is necessary, especially for gesture analysis, where spatial positioning plays a critical role.
- The estimation accuracy of the thumb and fingers, which are important in gesture research, has been less well validated than that of many other joints that are considered to be easier to track (such as the wrist, elbow, and shoulder).
- Many studies used OpenPose. OpenPose remains a very popular method, and it is impossible to ignore when discussing the recent progress in HPE. However, considering the rapid progress of deep learning methods, OpenPose is no longer a state-of-the-art method.

In this study, we determined the optimal methods while addressing the above drawbacks of previous studies.

- Unlike prior studies that focused on gross motor tasks, this work targets a realistic scenario that is typical of those used for gesture research to emphasize their importance in gesture-space research.
- We provide the detailed evaluation of the estimation accuracy of the 13 keypoints of the upper body, including the fingers and face, which are the main targets of gesture research.
- We compared MoCap with the 3D positions of keypoints estimated by four recent methods based on HPE. These four methods comprise two measurement methods applied to two leading HPE methods. The first HPE method, which focuses on accuracy, is the state-of-the-art method. The second method, which focuses on simplicity, is very easy to use and does not even require a graphics processing unit (GPU), which is required for most deep learning methods. The first measurement method estimates 3D positions using only a single viewpoint and the abilities of deep learning. The second method estimates 3D positions from two viewpoints using classical triangulation. Although deep learning methods can estimate the 3D positions of keypoints even from a single viewpoint if they are trained on a large amount of data, the accuracy of such estimates is often limited. Hence, we included the classical triangulation method in the comparison.

We know that the keypoints can be measured by a depth sensor such as the Microsoft Kinect, which was the mainstream method for 3D keypoint estimation before the recent development of deep learning. More communicative and less communicative groups were compared with respect to several kinematic features measured by the Kinect [3,4]. Because the authors reported that they did not expect the Kinect to replace manual annotation, this suggests that the accuracy of the Kinect had room for improvement. The trajectories of the keypoints [20], range of motion of joints [2], and gait parameters [5] measured by the Kinect were compared with those of MoCap for several simple tasks. The results showed that the accuracy of the keypoint positions would be insufficient for our purposes, but that of the gait parameters could be useful to some extent. Dance movements have been reconstructed in 3D with high accuracy using six Kinects [21]. However, this approach requires a complex setup and is technically challenging for researchers outside of the field of computer vision. MoCap based on inertial measurement units (IMUs) is also available, but it is still expensive and requires the user to attach the IMUs. Considering that the HPE code could be provided as a cloud service, video-based HPE would be easier for gesture researchers to use. Since the progress of HPE to date suggests that its accuracy will continue to improve, deep learning-based HPE is poised to remain the dominant trend in markerless MoCap.

## Materials and methods

### Participants and datasets

Ten adults (mean age = 22.1, standard deviation = 0.83, range = 21–24; 4 males) participated in the experiment. Participants were prospectively recruited for this study between July 4, 2023 and July 21, 2023. They were all native Japanese speakers. The research was approved by the Research Ethics Committee of University of Tsukuba (2023R737, 2025R005), and written informed consent was obtained from each participant. The individuals pictured in this manuscript have given written informed consent (as outlined in PLOS consent form) to publish these case details.

**Fig 1:**
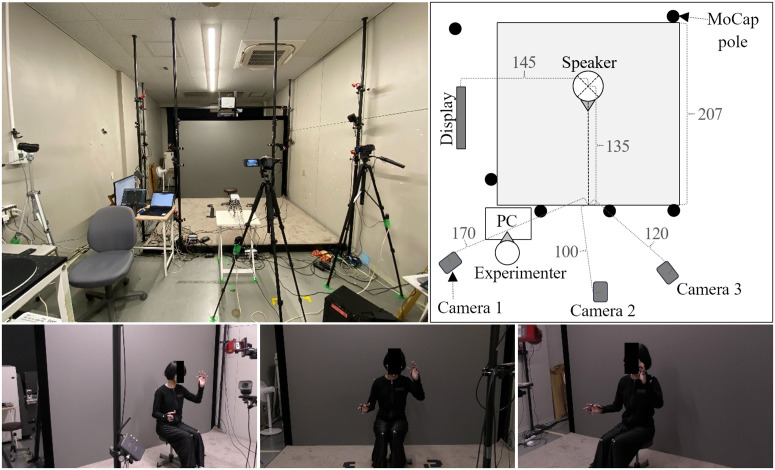
Experimental setup. The experimental room (top left), top view of the room (top right), and experimental video captured by the three cameras (second row).

After viewing an animated video (*Tweety*, Warner Bros.) of about one minute duration twice on a display placed outside the MoCap measurement space on the participant’s right side, the participant was asked to describe the video content while facing the front camera. The participant was instructed to use as many gestures as possible. Eight animated videos were used for data collection, resulting in a total of 80 data sequences (10 participants × 8 videos). As each sequence was simultaneously recorded by three video cameras, a total of 240 video files were collected for the analysis. Before the start and after the end of each participant’s description, a clapperboard with retroreflective markers was struck so that the duration of each data sequence could be manually adjusted. The participants were captured by a MoCap (OptiTrack Flex 3), and three video cameras (Panasonic HC-VX992MS: one set directly in front of the participant and two diagonally in front). Fig 1 shows the experimental setup and example images of a participant captured by the three cameras. All videos were recorded at a resolution of 1920×1080 with a frame rate of 30 frames per second (fps). After the cameras were focused on the participant, autofocus was disabled to ensure that the calibration accuracy was not affected by focusing [22].

### Pose estimation methods

Four methods were compared with MoCap in this study. To ensure that HPE was represented by the most accurate HPE method, the recently released RTMPose (hereafter RTM) was chosen [23]. RTM is reported to be more accurate than many methods, including OpenPose, and runs at very high speed on an inexpensive GPU. RTM is based on MMPose, an open-source toolbox for pose estimation; we used version 1.1.0 [24]. RTM estimates the 2D position and confidence value of 133 keypoints (17 for the body, 6 for the feet, 68 for the face, and 21 for each hand). The confidence value (between 0 and 1) is an indicator, provided by the model itself, of how likely the prediction is to be correct. This confidence value may decrease when keypoints are occluded. Model sizes were “M” for the detection and “X” for the pose estimation. RTM ran at a speed of about 2.1 fps in our implementation. The video was captured by a camera placed directly in front of the participant (camera 2 in Fig 1). RTMPose by itself can only detect 2D keypoints. Therefore, we used MocapNET (version 2) [25]. MocapNET is a method that converts 2D input keypoints into 3D keypoints using a model that is trained on the correspondence between 2D and 3D keypoints. The combination of RTMPose and MocapNET (hereafter referred to as RTM-mono) can estimate 3D keypoints from only one viewpoint. MocapNET converted 133 keypoints to 212 keypoints (19 for the body, 40 for the feet, 99 for the face, and 27 for each hand). In our case, it took only a few tens of seconds to run MocapNET on a single video.

**Fig 2:**
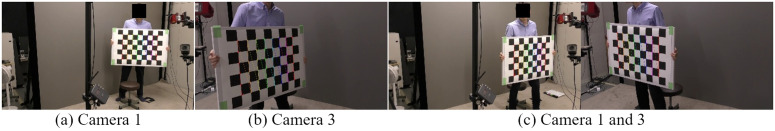
Checkerboard pattern examples. **(a)** An example frame from the video sequence used for the intrinsic calibration of camera 1. Many such frames, capturing the checkerboard from various angles, are used to determine the camera’s internal parameters like focal length and lens distortion. **(b)** An example frame for the intrinsic calibration of camera 3. **(c)** A synchronized pair of frames from cameras 1 and 3, used for extrinsic calibration. This process determines the precise 3D position and orientation of one camera relative to the other.

Despite recent developments in deep learning, the estimation of 3D keypoints from only one viewpoint is not sufficiently accurate. Therefore, we also implemented a method to estimate the 3D keypoints from 2D keypoints by triangulation using images from two video cameras. Triangulation is a classic computer vision technique that requires two calibrated video cameras. For the calibration, we used a common checkerboard pattern [26]. From a 30-s to 60-s video capturing a checkerboard pattern from various angles, every third frame was extracted. All frames in which the checkerboard was correctly detected among the extracted frames were used to calculate the intrinsic parameters of cameras 1 and 3 as well as the extrinsic parameters of camera 3 relative to camera 1 (note that the extrinsic parameters of camera 1 are defined such that the rotation is the identity matrix and the translation is the zero vector). The experiment was conducted over three days. However, because the video cameras became misaligned between the second and third days, calibration was performed again on the third day. Fig 2 shows an example of frames used for the calibration, and Table 1 presents the number of frames and the re-projection errors for each parameter. This combination of RTM and triangulation is referred to as RTM-tri. All calibration code was implemented using OpenCV (Python wrapper) 4.1.2.30. The confidence value in RTM-tri was set to the lower of the two confidence values in the RTM-mono outputs from the two cameras. This is because, if a keypoint is visible from one camera (high confidence value) but not from the other (low confidence value), the accuracy of the estimated 3D position is expected to be low. Triangulation used two videos from the cameras placed diagonally in front of the participant (cameras 1 and 3 in Fig 1).

**Table 1:** Calibration frame counts and re-projection errors for each parameter. The number of frames used to compute intrinsic and extrinsic parameters for cameras 1 and 3, along with the corresponding re-projection errors in pixels.

	No. frames / Error (pixels)	
Camera ID	1	3
Intrinsic (Days 1 & 2)	254 / 0.42	268 / 0.58
Intrinsic (Day 3)	367 / 0.57	294 / 0.90
Extrinsic (Days 1 & 2)	–	271 / 1.05
Extrinsic (Day 3)	–	250 / 1.63

MediaPipe Pose (MP; version 0.9) was chosen for further comparison [27]. Although MediaPipe uses deep learning models for keypoint detection, it is more accurately described as a framework that incorporates these models into a broader pipeline alongside heuristic-based components, such as graph optimization. MP is useful for gesture research because little code is required to implement it and it achieves near-real-time performance even without GPUs. The benefits of MP have also been reported in an occupational therapy study [28]. In addition, it is possible to perform 3D keypoint estimation from a single RGB image. MP estimates the 2D position of 543 keypoints (33 for the body, 468 for the face, and 21 for each hand). MP can also estimate a confidence value, but only for the body keypoints. For other keypoints, the confidence value is always set to 1 if the keypoint is detected. Of the three types of models available for MP, the most complex (accurate but slow) model was used. MP-mono and MP-tri, both based on MP, were implemented; these are analogous to RTM-mono and RTM-tri, which are based on RTM. MP-mono and MP-tri ran at about 9.7 and 4.8 fps, respectively, in our implementation.

**Fig 3:**
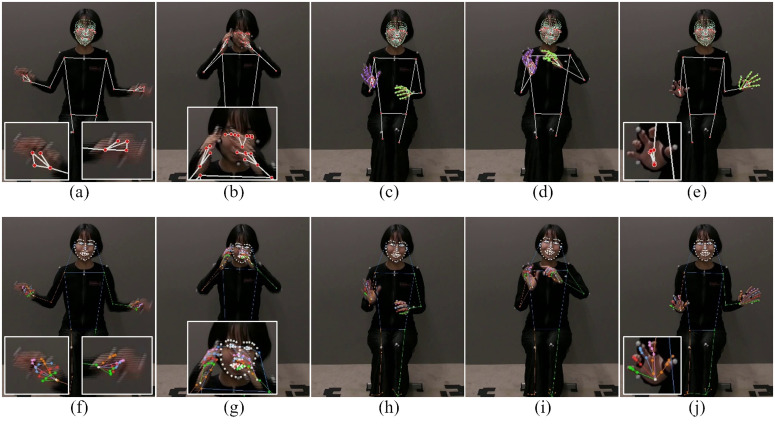
Examples of pose estimation results. Results for MP (first row) and RTM (second row). The images are of a participant (ID 5, who is describing the content of the first animation) taken by the camera placed directly in front of the participant. Some parts of the images are enlarged for better viewing.

Fig 3 shows examples of the MP and RTM results. The images reveal that both methods achieve very high accuracy, at least for 2D keypoint estimation. However, MP often failed to estimate keypoints, especially in the face (Fig 3b) and hands (Fig 3a, 3b, 3e). By contrast, RTM had fewer such cases (Fig 3f, 3g, 3j).

For the MoCap recordings, 19 retroreflective markers were attached to the participant: on the tips of the toes, knees, thumbs, middle fingers, and bases of the little fingers as well as on the wrists, elbows, shoulders, cheeks, and chin. For one participant (ID 1) only, the marker was attached to the earlobes instead of the cheeks. The MoCap system was calibrated daily using its calibration system during the three days of dataset recording. Because the software we used could not automatically determine which retroreflective marker corresponded to which keypoint, the annotations were performed manually while checking the viewer. MoCap occasionally failed to track the markers, causing the labels of the annotated keypoints to be lost. To avoid the cost of manual labor, we did not relabel these lost labels. Instead, keypoints that were lost once were treated as detection failures and excluded from further analysis. Since the purpose of this study was to compare MoCap and HPE, these partial MoCap measurement failures are not problematic. MoCap does not estimate the confidence values of keypoints; instead, it just omits keypoint positions if the measurement fails. The MoCap system ran at 100 fps.

The experiments were conducted on a computer with an Intel(R) Xeon(R) W-2223 CPU at 3.60 GHz and a GeForce RTX 3090 (24 GB). The operating system was Ubuntu 18.04. All code was implemented in Python.

### Data alignment

To compare the 3D keypoints estimated by HPE methods with those measured by MoCap, it was necessary to align the keypoints in time and space. We chose the 13 keypoints of the upper body, which are often the main targets of gesture research. These keypoints include the tips of the left and right thumbs and middle fingers, wrists, elbows, shoulders, cheeks, and chin. For each HPE method, if there was no keypoint that represented exactly the same position, the closest keypoint was selected instead.

The data captured on each device were temporally aligned at the frame level according to the clapperboard. To ensure accuracy, this manual annotation was performed by precisely identifying the exact video frame of the clapperboard’s impact and cross-referencing it with the corresponding peak in the audio waveform. For the MoCap, the images were not captured, so the start and end frames were manually annotated by visually checking the positions of the retroreflective markers. Of the three video cameras, only the one directly in front of the participant was manually annotated. Since the frame rates of the three video cameras were the same, the start and end frames of each data sequence of the remaining two cameras were automatically calculated, with the exception of the start frame of the first data sequence. After the annotation process, it was confirmed that the data from the three video cameras had the same number of frames. Note that the first and last five s of each data sequence were excluded from the analysis because the authors were visible in the frame to clap the clapperboard. The frame rates of the video cameras were lower than that of the MoCap system, and hence the number of MoCap frames was made equal to that of the video cameras after all start and end frames were aligned.

Because the devices had different coordinate systems, these also needed to be aligned. A rigid transformation was used for this purpose [29], as affine transformation can cause unnatural deformation of the keypoints and lead to falsely high accuracy. In RTM-mono and MP-mono, the scale cannot be defined. Hence, to ensure the scale was uniform, the distance between the right elbow and shoulder of each participant was measured with a tape measure. To ensure consistency among participants, this measurement was taken from the acromion (shoulder) to olecranon (elbow) on each participant’s right arm. Then, prior to the rigid transformation, scaling was performed so that the estimated distance between the right elbow and shoulder in RTM-mono and MP-mono matched the distance measured by the tape measure. The parameters of the rigid transformation and the scaling were computed from valid keypoints for each participant. These valid keypoints were extracted from all frames of the eight videos of each participant and were those that were judged to be valid by all methods; that is, keypoints detected by MoCap and detected with a confidence value at or above a certain threshold (0.3 in this study) by the HPE methods. The distance between the elbow and shoulder was calculated only when both of these keypoints were judged to be valid, and the mean value was used for the scaling.

## Results

**Fig 4:**
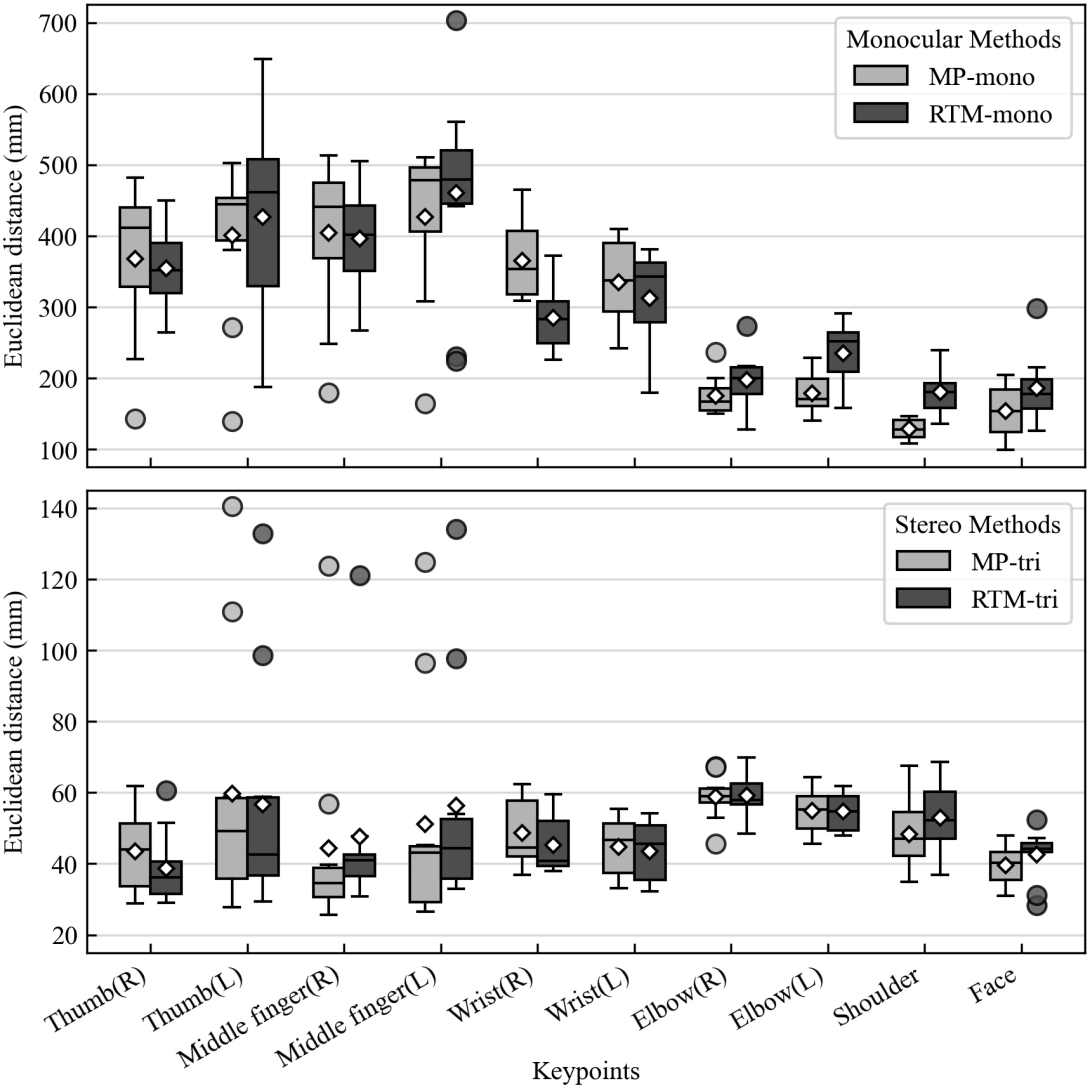
Distribution of Euclidean distance errors for each keypoint across participants. Each box plot visualizes the distribution of mean errors from the 10 participants. Euclidean distance is the difference between a keypoint measured by MoCap and that measured by another method. The diamond shows the average, the central line indicates the median, the box represents the interquartile range (IQR; 25th-75th percentiles), and the whiskers extend to the furthest data points within 1.5 times the IQR from the box. Data points beyond this range are considered outliers and are plotted individually. **(R)**: right side of the participants; **(L)**: left side of the participants.

As the error metric, the Euclidean distance was calculated for each keypoint for each participant with respect to MoCap (only for the valid keypoints, as described in the Data Alignment section). The two or three keypoints of the shoulder or face were analyzed together. Statistical analysis was then performed using Microsoft Excel and HAD statistical software [30]. The results are presented in Table 2 and Fig 4.

**Table 2:** Results of one-way ANOVAs. The two or three keypoints of the shoulder or face were analyzed together. N: the total number of valid keypoints included in the 80 data sequences; (R): right side of the participants; (L): left side of the participants. Reported values include degrees of freedom, F values, p values, effect sizes, and 95% confidence intervals of the mean differences.

Keypoints	N	F value	Partial $\eta^{2}$	95% CL (mm)	Multiple comparisons
Thumb (R)	35,684	81.86^***^	0.90	[181.14, 221.04]	MP-tri < MP-mono, RTM-tri < MP-mono
					MP-tri < RTM-mono, RTM-tri < RTM-mono
Thumb (L)	46,095	51.27^***^	0.85	[199.46, 272.71]	MP-tri < MP-mono, RTM-tri < MP-mono
					MP-tri < RTM-mono, RTM-tri < RTM-mono
Middle	51,679	78.11^***^	0.90	[202.56, 243.52]	MP-tri < MP-mono, RTM-tri < MP-mono
finger (R)					MP-tri < RTM-mono, RTM-tri < RTM-mono
Middle	55,477	62.26^***^	0.87	[211.93, 285.43]	MP-tri < MP-mono, RTM-tri < MP-mono
finger (L)					MP-tri < RTM-mono, RTM-tri < RTM-mono
					MP-tri < RTM-tri
Wrist (R)	128,650	262.32^***^	0.97	[170.47, 201.64]	MP-tri < MP-mono, RTM-tri < MP-mono
					MP-tri < RTM-mono, RTM-tri < RTM-mono
					RTM-mono<MP-mono, RTM-tri < MP-tri
Wrist (L)	132,269	123.63^***^	0.93	[166.46, 201.41]	MP-tri < MP-mono, RTM-tri < MP-mono
					MP-tri < RTM-mono, RTM-tri < RTM-mono
Elbow (R)	127,956	102.51^***^	0.92	[112.65, 132.27]	MP-tri < MP-mono, RTM-tri < MP-mono
					MP-tri < RTM-mono, RTM-tri < RTM-mono
Elbow (L)	142,859	131.20^***^	0.94	[120.40, 141.29]	MP-tri < MP-mono, RTM-tri < MP-mono
					MP-tri < RTM-mono, RTM-tri < RTM-mono
					MP-mono<RTM-mono
Shoulder	278,455	140.69^***^	0.94	[95.22, 109.91]	MP-tri < MP-mono, RTM-tri < MP-mono
					MP-tri < RTM-mono, RTM-tri < RTM-mono
					MP-mono<RTM-mono, MP-tri < RTM-tri
Face	396,990	72.43^***^	0.89	[91.68, 118.90]	MP-tri < MP-mono, RTM-tri < MP-mono
					MP-tri < RTM-mono, RTM-tri < RTM-mono

^***^p < 0.01. For all keypoints, df1 = 3 and df2 = 27.

We conducted several one-way analyses of variance (ANOVAs) to test whether the Euclidean distances differed among the four HPE methods. We used the method type as a within-subject independent variable and the mean Euclidean distance for each keypoint as the dependent variable. The ANOVA results showed a main effect of method type for all keypoints, and p-values were less than .001 for all tests. The degrees of freedom, F values, p values, effect size, and 95% confidence intervals for the result of each keypoint are reported in Table 2. Bonferroni *t*-tests (*p* < .05) showed that both stereo methods had significantly smaller errors than both monocular methods for all keypoints. By contrast, the accuracies of MP and RTM were similar. The distance of MP-tri was significantly smaller than that of RTM-tri for the left middle finger/shoulder keypoints, and the distance of MP-mono was significantly smaller than that of RTM-mono for the left elbow/shoulder keypoints. Moreover, RTM-tri and RTM-mono had significantly smaller distances than MP-tri and MP-mono, respectively, for the right wrist keypoint.

**Fig 5:**
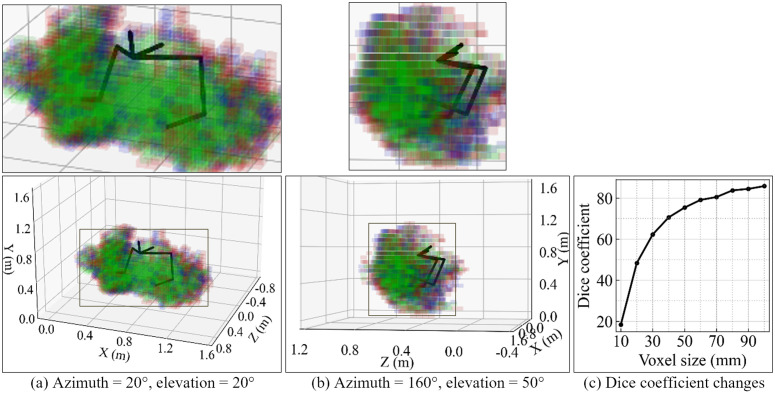
Visualization of the gesture space. The gesture space is visualized with a voxel size of 50 mm, showing the same data from two perspectives: (a) a front-left-above view (azimuth = 20°, elevation = 20°) and (b) a left-rear-side view (azimuth = 160°, elevation = 50°). When the azimuth is 0° and the elevation is 0°, the figure faces the front of the participant. Green voxels include both MoCap and RTM-tri observations, and red or blue voxels include only MoCap or RTM-tri observations. The central parts of the images are shown enlarged at the top for better viewing. The mean positions of the participant’s keypoints measured by MoCap are plotted with solid lines. All data sequences from participant ID 1 were used. The Dice coefficient at a voxel size of 50 mm was 75.4%. **(c)** Changes in the Dice coefficient associated with changes in the voxel size.

We also compared the gesture spaces measured by MoCap and RTM-tri to evaluate the practicality of the HPE methods. In Fig 5 a and Fig 5b, the space was divided into a grid of 50-mm voxels, and whether the position of the left or right wrist was included at least once in each voxel is visualized (i.e., information about the left or right wrist and time is not considered). The dominant green voxels represent those containing both MoCap and RTM-tri observations. The Dice coefficient, which indicates the similarity of the sets, was 75.4%. Obviously, the larger the voxel size, the higher the agreement between MoCap and RTM-tri should be. Fig 5c shows the relationship between the voxel size and Dice coefficient. The Dice coefficient was very low (18.4%) when the voxel size was 10 mm, but exceeded 80% when the voxel size was 70 mm or larger.

## Discussion

### Validity and practicality of results

The overall average error of MP-tri was 49.4 mm, and that of RTM-tri was 49.8 mm. We note that the marker positions measured by MoCap and the keypoint positions measured by the HPE methods did not match (Fig 3). This is because the markers were attached with reference to the bone positions, and hence they were placed at the same positions for all participants, whereas the HPE methods detect keypoints inside the body, not on the bones. Moreover, the markers can only be attached to the skin (outside the body). In other words, the marker positions change depending on the body orientation, but the 2D positions of the HPE keypoints hardly change because of the body orientation alone. Therefore, it is not possible to strictly match the marker and HPE keypoint positions. To contextualize these results, we also report confidence intervals for the key comparisons (Table 2), providing a clearer indication of variability across participants. We note that gesture research has traditionally relied on visual observation and manual annotation, both of which involve substantial inter-observer variability. Additionally, since the average adult hand breadth is approximately 75–85 mm [31,32], an average error of less than 50 mm is roughly half the average hand breadth. From these perspectives, an average error of approximately 50 mm is unlikely to affect categorical distinctions between gesture space regions (e.g., left vs. right or upper vs. lower space). Furthermore, the required accuracy of keypoint position estimation depends on the research goals or applications. In the context of gesture research, where the focus is often on identifying the spatial extent, distribution, or dynamics of manual movements [6], extreme precision down to millimeters may not always be necessary. Considering the above, we believe that an error of less than 50 mm is acceptable. Nonetheless, future research should systematically examine correlations between measurement error and the precision of gesture interpretation to establish thresholds for meaningful spatial variation.

The error distribution shown in the box plots (Fig 4) reveals another important detail: even the highly accurate stereo methods (MP-tri and RTM-tri) produced several large-error outliers for the thumb and middle finger keypoints. This suggests that while the median accuracy for fingers is high, their small size, rapid movements, and frequent self-occlusion make them susceptible to tracking failures, resulting in these outliers. In contrast, larger keypoints (wrists, shoulders, and elbows) exhibit a more stable error distribution with fewer outliers.

Our findings demonstrate that, despite its lower spatial resolution compared to marker-based MoCap systems, the HPE methods provide sufficiently accurate 3D position data to capture meaningful patterns in gesture production. Specifically, we demonstrated that HPE is capable of detecting systematic spatial tendencies in gesture trajectories, such as lateralisation, vertical reach, or proximity to the body, that are central to the study of gesture space (e.g., [6]). This capability allows gesture space to be examined not only qualitatively or in two dimensions, but also quantified in 3D space in an automated and scalable manner. Such affordances open up new possibilities for analysing gesture-speech alignment, cross-linguistic variation in gesture form, and even individual differences in gestural style using computational methods. In this sense, HPE offers a promising and accessible alternative to MoCap, especially for researchers who wish to explore gesture space as a measurable construct but lack access to specialised hardware. As deep learning-based methods continue to improve, the ability to estimate and model the volume of space in which gestures are produced can contribute to a deeper understanding of how gestures are shaped by cognitive, communicative, and cultural factors.

Overall, our results show that video cameras and general computing resources can be a substitute for MoCap for purposes such as those mentioned above. Video cameras are much cheaper than MoCap systems, and the widespread availability of smartphones makes them the most readily available device. In our experiments, we had to place the video cameras several meters away from the participants (Fig 1) because of the narrow field of view of the video cameras, but they are quite easy to place and remove, and they do not require a dedicated space. The fact that no retroreflective markers must be attached to the participants is also convenient for gesture researchers. HPE can detect a large number of keypoints (for example, 543 keypoints for MP), which should be sufficient for many gesture research applications. The frame rate is also flexible because it depends on the frame rate of a video camera, although processing time is longer for higher frame rates.

### Performance analysis of individual HPE methods

**Fig 6:**
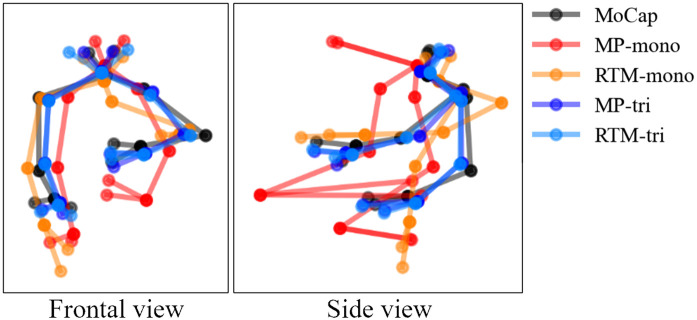
Comparison of MoCap with the four HPE methods. The keypoint positions estimated by each method for participant ID 5 are shown in different colors at the frame from the fifth animation.

The experimental results showed that the monocular methods had quite large errors (Fig 4). This is because the error in the depth direction was very large, even though the 2D keypoint positions of the participants’ frontal faces used for the estimation were accurate. Fig 6 shows that discrepancies between the monocular methods (MP-mono and RTM-mono) and MoCap are particularly pronounced in the side view; that is, in the depth direction from the camera’s perspective. We found that depth estimation from a single view is still difficult, even with recent advances in deep learning.

In terms of positional accuracy on successfully detected keypoints, MP-tri and RTM-tri were very similar. However, RTM-tri was superior from another perspective. Table 3 shows the percentage of estimated keypoints with a confidence value below the threshold (0.3). RTM-tri was able to estimate keypoints with higher confidence quite often. This was particularly evident for the thumb, middle finger and face keypoints. The high failure rate of MP-tri is a significant practical limitation. This finding does not compromise our accuracy comparison, which is validated for the subset of successfully detected keypoints, but it does highlight a key weakness of MP-tri. This is consistent with the qualitative results (Fig 3). MP-tri seemed to abandon estimation in difficult cases instead of forcing an estimate (Fig 3a, 3b, 3e). By contrast, RTM-tri had some cases of false positives (e.g., detecting a face where there is no person, although there were no such cases in the dataset used in this study). Although the positional accuracies on successfully detected keypoints were similar, RTM-tri’s superior detection reliability (Table 3) establishes it as a more robust method overall. However, MP has the significant practical advantage of being easy to implement and not requiring a GPU.

**Table 3:** Percentage of keypoints with a confidence value below 0.3, either in RTM-tri only, MP-tri only, or both (%). “N” is the total number of low-confidence keypoints. Shoulder keypoints were detected in all frames.

	N	RTM-tri	MP-tri	Both
Thumb (R)	57,952	0.12	97.73	2.15
Thumb (L)	47,266	0.17	95.81	4.02
Middle finger (R)	57,947	0.11	97.63	2.26
Middle finger (L)	47,270	0.18	95.55	4.28
Wrist (R)	1,173	35.55	55.24	9.21
Wrist (L)	1,787	26.36	68.33	5.32
Elbow (R)	1,230	21.22	78.70	0.08
Elbow (L)	1,585	17.48	81.07	1.45
Shoulder	0	NA	NA	NA
Face	18,295	0.41	99.59	0.00

### Limitations and future directions

This study has several limitations. The first category of limitations relates to the measurement error. There was an anatomical mismatch between our MoCap ground truth and the HPE estimations. This discrepancy contributes an unquantified amount of error to the 50 mm average error and impacts the interpretation of absolute accuracy. Keypoints with more surrounding soft tissue (e.g., shoulders) should be more susceptible to this mismatch than keypoints like fingertips. Separating this anatomical error from the algorithmic estimation error is a significant technical challenge. A related limitation applies to the scaling procedure required for the monocular methods. This process relied on a physical, external measurement of the bone-to-bone distance between the acromion (shoulder) and olecranon (elbow). This externally measured distance does not perfectly correspond to the distance between the internally inferred keypoint centers of the HPE models. This discrepancy introduces a potential source of scaling error that is specific to the monocular results. There are also discrepancies between HPE models. Comparing keypoints from different models that may have subtle differences in their trained definitions introduces another potential source of bias. Although we carefully confirmed the correspondence of the keypoints through visual inspection, minor discrepancies may exist, especially when mapping a dense set of keypoints to a sparse set (e.g., mapping MocapNET’s 99 facial points to the RTM-mono condition). While we believe this effect to be minimal, it remains a limitation of this cross-model comparison. Finally, beyond spatial accuracy, our temporal alignment exhibited two limitations. The synchronization of the videos relied on careful manual annotation of the clapperboard signal. While we believe our method to be accurate, automated synchronization methods could offer greater precision and reliability. Another limitation lies in aligning the MoCap and video data. We synchronized the start and end of each trial, then downsampled the 100 Hz MoCap data to match the video frame rate uniformly. We considered this approach sufficient for the relatively low-velocity conversational gestures in our study, and a visual inspection confirmed the absence of significant motion artifacts. However, this downsampling process introduces a risk of minor phase misalignment between the two data streams. Future work requiring higher temporal precision would benefit from recording video at a higher frame rate or matching the sampling rates of both systems before the data acquisition.

The second category of limitations relates to the generalizability of our findings. Our participant sample was small (N = 10) and demographically homogeneous (young Japanese adults). Since body proportions and gesture styles can vary across different ages, cultures, and body types, the accuracy results reported here may not be applicable to other populations. The generalizability is also affected by our choice of models. We selected RTMPose and MediaPipe Pose as two representative HPE methods that span the spectrum from high accessibility to state-of-the-art performance, considering the needs of gesture researchers. MediaPipe was chosen for its exceptional ease of use, minimal setup requirements, and ability to run efficiently without a dedicated GPU, making it a practical choice for non-experts. In contrast, RTMPose was chosen to represent the current state-of-the-art in accuracy, superseding older baselines like OpenPose. Other powerful methods exist. Some, such as VIBE [33] and HybrIK [34], focus on 3D mesh and shape recovery rather than the direct keypoint coordinate estimation, which is central to this study. While we are aware that HPE, as developed by computer vision researchers, has been criticized for its lack of biomechanical knowledge [35], it still has many advantages when the required accuracy is not as high as that of MoCap, as noted above. Furthermore, this study did not investigate the optimal camera arrangement (e.g., resolution, number, distance) which is another practical factor that will influence performance like other studies [36,37].

Addressing these limitations provides a clear roadmap for future research aimed at developing a truly robust and universally applicable system for gesture analysis.

## Conclusions

This study evaluated the accuracy and feasibility of four recent deep learning-based 3D HPE methods as alternatives to optical motion capture (MoCap) for gesture research by comparing 3D upper-body keypoints during gesture-rich speech. We found that stereo methods outperformed monocular methods across all keypoints. The most accurate stereo HPE method achieved an average error of 49.4 mm, which is acceptable given the inherent differences in the keypoint definitions of HPE and MoCap. These findings demonstrate that stereo HPE is a viable, cost-effective alternative to MoCap for researchers seeking 3D gesture data, particularly in settings with limited access to specialized equipment. In addition to examining keypoint accuracy, we examined spatial overlap in gesture space and found a 75.4% voxel-wise agreement between HPE and MoCap when using a 50-mm voxel. This further supports the practical utility of HPE in capturing gesture dynamics in 3D space.

Our future goal is to develop and release a highly accurate toolbox for 3D HPE that is easy for gesture researchers who are not experts in machine learning to operate. Specifically, we plan for this toolbox to include codes that can be run on a cloud-based interface for camera calibration and pose estimation. The toolbox will output 3D coordinate data in accessible formats. This study lays the groundwork for a user-friendly 3D gesture analysis toolbox aimed at non-expert researchers in psychology, linguistics, and communication sciences. In addition, study was intended mainly for gesture research; however, its results are also relevant to the development of practical interfaces such as remote calls and interactive AI, in addition to its contributions in research fields such as psychology and linguistics.
